# The United States of America and the Islamic Republic of Iran: the path to preventing traffic injuries?

**DOI:** 10.1186/1478-7954-8-4

**Published:** 2010-03-31

**Authors:** Ali H Mokdad

**Affiliations:** 1 Institute for Health Metrics and Evaluation, University of Washington, 2301 5th Ave, Suite 600, Seattle, WA 98121, USA

## 

Reducing and preventing road traffic injuries (RTIs) is a challenge faced by all nations [1]. The manuscript by Naghavi et al. highlights the huge burden of injuries in Iranian children [2], most of which are the result of road traffic injuries. The manuscript revealed a rising death rate from injuries among children from 1971 to 2005. The authors' previous work has documented the fact that Iran has the highest RTI death rate of any country where such data are available [3,4]. The authors described and documented the main reasons for the rapid increase in RTIs. It is not a surprise that Iran's subsidized gas, which is 10 times cheaper than its production cost, and the rapid increase in vehicle production (more than 1 million cars and 1.5 million motorcycles produced every year since 2002) are important contributing factors in Iran's RTI burden [4]. Indeed, when it comes to RTIs, Iran is experiencing one of the downsides of joining the club of countries with rapid economic growth over the past three decades.

The manuscript has some limitations. The comparison to previous years is weakened by the limited amount of historical data. Only data on the capital Tehran were available in 1971. In addition, improvements in the quality of data throughout the study period make the data difficult to compare to previous years. The most important limitation is the completeness of the data and whether the increases in RTI rates are due to better statistics about RTIs rather than true rates of increase. As much as the latter is true, it still does not explain all the increases in RTIs in Iran. However, the results of the study and the previous work of the authors provide a clear picture of RTIs in Iran today and their increasing burden. In general, the study shows the importance of the data systems that capture the burden of injuries in Iran and suggests such systems could be a model for other countries with similar economic growth.

Strategies to prevent traffic injuries have been discussed and promoted in many publications [1,5]. These measures include designing safer cars and roads and enforcement of speed and weight limits. In addition, enforcing occupant protection through child safety seats, seat belts, and helmet use, to name a few, is essential. Unfortunately, Iran has a long way to go to reach these objectives. Iran is a country with deep cultural roots; however, it is a late bloomer when it comes to modern mechanization. Iran's rapid increase in motor vehicle use may have started in the 1950s, with its first automobile production occurring in the late 1960s. Car production started its peak in 2002 [4]. This would place Iran about 80 years behind the US and much of Europe in motor vehicle use.

What Iran is facing now is similar in many ways to what the US once faced. At the beginning of the century, motor travel in the US was a novelty, with an estimated 8,000 automobiles on the road. By 2000, more than 226 million vehicles were registered [6]. As the number of vehicles and drivers in the US increased, so did deaths and injuries - from 1.0 motor-vehicle deaths per 100,000 to 26.7 per 100,000 in 1930 [6]. For the most part, these rates remained very high until 1970 (27.7 per 100,000), except for a brief decline during World War II due to lack of fuel availability [6]. After 1970, rates began to decline. It took the US about 70 years to curb the burden of RTIs. Iran and other countries should not follow this model.

The victories of motor vehicle safety in the US are well-documented [7]. Substantial gains in driver education, vehicle safety, and road design were implemented through legislation and public awareness campaigns. Above all, these victories were due to the work of public safety champions. President Herbert Hoover convened the first National Conference on Street and Highway Safety in 1924. President Franklin D. Roosevelt convened an Accident Prevention Conference on vehicle safety in 1936 and called for lower speeds, better lighting, and stronger auto frames. President Lyndon B. Johnson signed the National Traffic and Motor Safety Act and the Highway Safety Act in 1966. Lawyer and activist Ralph Nader led a consumer movement for vehicle safety in the 1960s. His work inspired hundreds of activists who joined together to help with his work (they came to be known as "Nader's raiders"). William Haddon, a public health physician and epidemiologist who became the first director of the National Highway Safety Bureau, revolutionized the scientific approach to preventing motor-vehicle injuries by developing the Haddon Matrix [8]. His work recognized that injuries are like infectious diseases, a result of the interaction between a host (person), an agent (motor vehicle), and the environment (road).

The US success story was built upon the hard work of champions who pushed to reduce RTIs and to improve motor-vehicle safety. As in many success stories, many components must come together to achieve the goal of safer roads. However, the essential ingredient is the dedication and vision of the champions who make motor-vehicle safety their cause. Iran is in dire need of champions such as those who have driven road safety improvements in the US. Their contributions will no doubt expedite the time it takes to achieve a success story similar to what the US has experienced. It is time for champions of RTI prevention and control and motor-vehicle safety to step forward in Iran. The manuscript by Naghavi et al. makes a strong case for Iran to follow the US's path, but at much faster pace.
